# Status of selected nutrients in obese dogs undergoing caloric restriction

**DOI:** 10.1186/1746-6148-9-219

**Published:** 2013-10-24

**Authors:** Deborah E Linder, Lisa M Freeman, Shelley L Holden, Vincent Biourge, Alexander J German

**Affiliations:** 1 Tufts Obesity Clinic for Animals, Department of Clinical Sciences, Cummings School of Veterinary Medicine, Tufts University, 200 Westboro Road, North Grafton, MA 01536, USA; 2 Department of Obesity and Endocrinology, University of Liverpool, Leahurst Campus, Chester High Road, Neston, Wirral CH64 7TE, UK; 3 Royal Canin Research Centre, Aimargues 30470, France

**Keywords:** Malnutrition, Overweight, Canine, Weight loss, Weight management

## Abstract

**Background:**

The purpose of this study was to test the hypothesis that dog plasma concentrations of selected nutrients decrease after undergoing caloric restriction for weight loss. Thirty-one overweight dogs that had successfully lost at least 15% of initial body weight were included in the study. Nutrients that had been previously identified to be at potential risk of deficiency during caloric restriction were measured in plasma (choline, amino acids) and urine (selenium) at the initiation and completion of a standardized weight loss regimen in dogs.

**Results:**

Dogs remained healthy throughout the study, and no signs attributable to nutrient deficiency were noted. Percentage weight loss was 28.3% (16.0-40.1%) starting body weight, over a period of 250 days (91–674 days). Median energy intake during the weight loss period was 62 (44 to 74) Kcal/kg^0.75^ target weight per day. Choline (*P* = 0.046) and threonine (*P* = 0.02) decreased after weight loss. Glycine (*P* = 0.041), and urinary selenium:creatinine ratio (*P* = 0.006) both increased after weight loss. There were no other significant differences in plasma nutrient concentrations.

**Conclusions:**

Since concentrations of most measured nutrients did not change significantly, the data are not consistent with widespread nutrient deficiency in dogs undergoing caloric restriction using a diet formulated for weight loss. However, the significance of the decrease in plasma choline concentration requires further assessment.

## Background

Obesity is one of the most common health problems of dogs, with between 34-59% of the canine population in the United States and Europe currently overweight or obese [1,2]. The condition is associated with numerous diseases and a shorter lifespan [3-5]. With growing awareness of the consequences of obesity, weight loss plans may become more common in veterinary medicine.

Various strategies can be used for weight loss, but most require caloric restriction, adjusted for each individual, and consideration must be given to choice of diet. Starting points for caloric restriction can vary from resting energy requirement (RER; 70 kcal/kg^0.75^) for current weight, to RER for ideal weight, to a percentage of the maintenance energy requirement (MER) for current or ideal weight [6-8]. However, adjustments are commonly required, during the course of the regimen, in order to achieve a rate of weight loss which is safe, maximises owner compliance, and minimises loss of lean body mass. The exact level of restriction required can vary considerably (50-82% of maintenance energy requirement at target weight) and overweight dogs with low energy requirements may require considerable caloric restriction [9].

Given that most dogs and cats are fed commercial complete and balanced pet foods, the intake of all nutrients in the diet is decreased when calories are restricted. Thus, it is possible that intake of some nutrients might be suboptimal during weight loss, particularly in animals with low energy requirements that require significant caloric restriction to achieve successful weight loss. Although recommendations for optimal macronutrient composition can vary [10-12], an increased nutrient:calorie ratio compared to maintenance foods is common in most foods formulated for weight loss to account for this caloric restriction. However, optimal nutrient density and levels of various nutrients of concern have not yet been determined. A recent *in silico* study highlighted the potential for nutrient deficiencies when using a range of diets at current levels of energy restriction [13]. However, arguably, *in vivo* studies are a better way of determining the existence of nutrient deficiencies. This information is important to aid in the formulation of optimal nutrient profiles for weight loss diets.

The nutrient status of an animal can be measured in various ways, including tissue biopsy (e.g. liver biopsy for copper, muscle biopsy for taurine), measurement of the nutrient in either plasma or urine, and using proxy measures of nutritional adequacy (e.g. urinary methylmalonic acid for cobalamin status, erythrocyte glutathione peroxidase measurement for selenium status) [14]. Measurement of plasma nutrient concentrations, have long been used to determine nutrient status in many mammalian species. For humans, plasma amino acid measurement has been used to evaluate protein-calorie malnutrition (PCM) in third world countries [15] whilst, in cats, it was plasma measurement of taurine that helped to identify taurine deficiency in cats [16]. In the field of animal nutrition, plasma nutrient measurements have been used extensively to determine both whether and which essential amino acid is limiting in a diet [17-19]. In dogs, one of the first uses of plasma nutrient measurement was more than 50 years ago, when the rate-limiting amino acid for growth was determined in dogs using plasma amino acid concentrations in dogs eating various dietary proteins [20]. More recently, plasma taurine, cysteine, and methionine have also been used to assess canine dietary completeness [21]. Therefore, although not perfect, such methods have the advantage that the samples can be acquired non-invasively. As a result, the current study used urinary and plasma nutrient measurements to determine if the status of selected nutrients changes in dogs undergoing caloric restriction for weight loss.

## Methods

### Ethics statement

The study protocol adhered to the University of Liverpool Animal Ethics Guidelines, and was approved by the University of Liverpool Research Ethics Committee. Owners of all participating animals gave informed written consent.

### Study design

This was a non-randomised observational study assessing changes in circulating nutrient concentrations in a cohort of dogs with naturally-occurring obesity, and has been reported according to the Strengthening and Reporting of Observational Studies in Epidemiology statement guidelines [22].

### Animals

Dogs were referred to the Royal Canin Weight Management Clinic, University of Liverpool, UK, for investigation and management of obesity and associated disorders. Thirty-one dogs were recruited between February 2005 and August 2010, and those successfully losing weight had completed by January 2011. Eligibility criteria included confirmation of obesity (based upon body fat measurement by dual-energy X-ray absorptiometry; DEXA), required loss of at least 15% of starting body weight, and availability of sufficient surplus plasma and urine for analysis.

### Weight loss regimen

Full details of the weight loss regimen have been previously described [9,23]. Briefly, dogs were determined to be systemically well, and without significant abnormalities on complete blood count, serum biochemical analysis and urinalysis. Throughout weight loss, patients were weighed on electronic weigh scales (Soehnle Professional), which were regularly calibrated using test weights (Blake and Boughton Ltd.). Body composition was analysed by fan-beam DEXA (Lunar Prodigy Advance; GE Lunar), and results were used to estimate target weight [23]. Pre-weight loss total body composition results were used to estimate target weight [9,24]. Further, by comparing pre- and post-weight loss DEXA scan results, change in fat and lean mass could be estimated [9,25].

A weight management protocol was then initiated [9,23], using a high protein high fibre weight loss diet (Table 1), and fed according to manufacturer’s instructions. The initial food allocation for weight loss was determined by first estimating maintenance energy requirement (MER = 440 kJ [105 Kcal] × body weight [kg]^0.75^/day) [26] using the estimated target weight. The exact level of restriction for each dog was then individualised based upon gender and other factors (i.e. presence of associated diseases such as osteoarthritis and other orthopaedic disorders), and was typically between 50-60% of MER at target weight [23]. Owners also implemented lifestyle and activity alterations to assist in weight loss. Dogs were reweighed every 7–21 days and changes were made to the dietary plan if necessary [9,23].

**Table 1:** Average analysis of the diet used for weight loss in the study dogs

**Nutrient**	**Per Mcal**	**Per 100 g as fed**
Kcal/kg Metabolizable Energy	2900^*^	---
Crude protein (g)	104.0	30.2
Arginine (g)	5.4	1.6
Histidine (g)	2.0	0.6
Isoleucine (g)	3.8	1.1
Met and Cys (g)	3.6	1.0
Leucine (g)	7.7	2.2
Lysine (g)	4.1	1.2
Phe and Tyr (g)	9.6	2.8
Threonine (g)	3.3	1.0
Tryptophan (g)	0.9	0.3
Valine (g)	4.4	1.3
Total fat (g)	33.0	9.6
Linoleic acid (g)	7.3	2.1
Calcium (g)	3.1	0.9
Phosphorus (g)	2.4	0.7
Magnesium (g)	0.17	0.05
Sodium (g)	1.0	0.3
Potassium (g)	2.8	0.8
Chloride (g)	3.0	0.9
Iron (mg)	57.0	17.0
Copper (mg)	6.9	2.0
Zinc (mg)	69.0	20.0
Manganese (mg)	24.0	6.9
Selenium (mg)	0.05	0.01
Iodine (mg)	1.0	0.3
Vitamin A (IU)	6990	2001
Vitamin D3 (IU)	242	70
Vitamin E (IU)	276	80
Thiamin (mg)	9.0	2.6
Riboflavin (mg)	18.0	5.2
Pyridoxine (mg)	8.0	2.3
Niacin (mg)	56.0	16.2
Pantothenic acid (mg)	16.0	4.6
Cobalamin (mg)	0.06	0.02
Folic acid (mg)	1.5	0.4
Choline (mg)	860	249

^*^All nutrients listed on an energy basis.

In dogs that successfully reached target weight, detailed evaluation was conducted after the weight loss. At this stage, body weight was recorded, and body composition assessed by DEXA. The detailed re-evaluation assessment was not conducted in dogs failing to complete, either because they were euthanased (for unrelated reasons) before reaching target weight or were lost to follow-up.

### Laboratory analyses

All blood and urine samples were taken after a fast of at least 16 h, from each dog, both before initiating the weight loss programme and when target weight was successfully achieved. A complete blood count was performed using whole blood from EDTA tubes. Serum was analysed for a biochemistry profile and thyroid (T4) concentrations. Immediately after collection, heparinized samples were centrifuged, and the plasma was harvested. Plasma not required for biochemical analyses was frozen at −20°C until analysis, within 30 min of sample collection. Samples remained frozen until they were shipped on dry ice to the laboratories chosen for analyte measurement (see below).

### Choline and betaine metabolite measurements

Plasma concentrations of free choline and betaine, were quantified, by the Caudill Laboratory, Cornell University, USA, using liquid chromatography-tandem mass spectrometry according to methods of Holm and Wang [27,28] with modifications based on the instrumentation [29]. The intra- and inter-assay precision (coefficient of variation) of the method is <3%, based on in-house control materials.

### Plasma amino acid assessment

Amino acids were analyzed with a commercial amino acid analyser^g^ by the Amino Acid Laboratory, University of California Davis, USA. Methionine was not measured because previous studies have demonstrated that this amino acid is susceptible to oxidation during storage [30]. Cysteine was also not measured because this amino acid is susceptible to breakdown if not treated with sulfosalicylic acid immediately after sampling [31].

### Urinary selenium assessment

Urinary selenium measurements were made by the Food and Environmental Research Agency, York, UK. Aliquots (1 mL) of test sample plus certified reference materials were digested in nitric acid using quartz high pressure closed vessels and microwave heating. The digests were diluted with water containing propan-2-ol and internal standard (Rhodium standard, “Aristar” grade, 1000 ppm. VWR International Ltd.), prior to quantification by inductively coupled plasma-mass spectrometry, using collision cell run in hydrogen mode (7500ce ICPMS, Agilient Technologies). Reagent blanks and samples spiked with a known amount of each analyte were analysed with the test samples for recovery estimate purposes. Urinary selenium concentrations were adjusted for urinary creatinine excretion, measured by an automated biochemistry analyser (Kone Specific Supra biochemistry analyzer; Thermo Fisher Scientific, Inc.) and results were expressed as a ratio of selenium to creatinine.

### Data analysis

Data are expressed as median (range) except where indicated. Statistical analyses were performed with computer software (Stats Direct version 2.6.8, Stats Direct Ltd.), with the level of significance set at *P* < 0.05 for two-sided analyses. Given that there were no known previous studies examining changes in nutrient biomarkers in obese dogs undergoing weight loss, a meaningful power calculation was not possible. Instead, the number of dogs enrolled was based upon the prior experience from previous studies of plasma biomarkers from the same clinic [25,32,33].

The Shapiro-Wilk test was first used to assess all data sets, and parametric and non-parametric tests were used as appropriate. These included simple and multiple regression analysis, the signed ranks test, the Mann–Whitney *U* test, and Kendall’s rank correlation.

Differences in either plasma nutrient concentrations, clinical biochemistry parameters, or urine selenium:creatinine ratio prior to and after weight loss, were assessed with the Wilcoxon signed ranks test, and associations between changes in plasma analytes and clinical biochemistry parameters were assessed with Kendall’s rank correlation. Where significant differences were identified, linear regression was then used to determine factors that were associated. The outcome variable of interest was percentage change in the respective nutrient (i.e. {[nutrient]_PRE_–[nutrient]_POST_} ÷ [nutrient]_PRE_ × 100), while factors tested included signalment factors (e.g. age at enrolment, sex, breed group [retriever vs. not retriever]), baseline parameters (e.g. percentage body fat pre-weight-loss, starting body weight, lean tissue mass pre-weight loss [in kg]), and weight loss outcomes (duration of weight loss, percentage weight loss, energy intake during weight loss, percentage change in body fat mass, and percentage change in lean tissue mass). Percentage change in fat mass was calculated using the following formula: (fat mass [kg]_PRE_–fat mass [kg] _POST_) ÷ fat mass [kg]_PRE_ × 100 ); similarly, percentage change in fat mass was calculated using the following formula: (lean mass [kg]_PRE_–lean mass [kg] _POST_) ÷ lean mass [kg]_PRE_ × 100). Initially, simple linear regression was used. A multiple linear regression model was then constructed, which initially included any variables identified as *P* < 0.2 on univariable analysis. The model was subsequently refined by backwards-stepwise elimination of the least significant variable at each round, and variables were retained in the final model if they were significant (*P* < 0.05), or where removal lead to a notable reduction (e.g. ≥30%) in the overall fit of the model.

## Results

### Dogs and outcome of weight loss

During the period of recruitment, (February 2005 to August 2010), 140 new cases were referred to the clinic; of these dogs, 79 dogs completed their weight loss program by June 2011, 4 were successfully losing weight but had not completed by June 2011, 7 dogs had died or been euthanased (for unrelated reasons) and the weight loss program had been discontinued in the remaining 40 dogs (either due to owner-related reasons including illness and bereavement, or because the owner chose to stop). Of the 79 dogs successfully reaching target, 31 were ultimately identified that had lost >15% body weight and had sufficient plasma and urine available for all assays to be conducted. During shipment, the tubes containing plasma samples from 5 of the 31 dogs were damaged and, as a result, only 26 dogs had plasma amino acids and choline performed. Paired sampled from 20 dogs were available for urine selenium analysis. Those 20 samples were selected from the original 31 dogs in order of percentage weight lost (i.e., the 20 dogs with the greatest percentage of weight loss had their samples analysed). Weight loss characteristics for dogs included in the study did not differ from those also completing but not ultimately being included in the study (data not shown).

In the 31 dogs finally included in the study, percentage weight loss was 28.3% (16.0-40.1%) starting body weight (SBW), over a period of 250 days (91–674 days); therefore, median rate of weight loss was 0.8% (0.3-1.4%) SBW/week (Table 2). Neither the signalment nor weight loss outcomes differed between the 26 dogs having choline and amino acid analysis, and those where it was not measured (*P* > 0.12 for all; data not shown); similarly, signalment and weight loss outcomes did not differ between the 20 dogs where urinary selenium was measured and those where it was not measured (*P* > 0.28 for all, data not shown).

**Table 2:** Summary of weight loss in the study dogs

***Criterion***	**Result**
*Age (months)*	66 (12 to 132)
*Sex*	17 NM, 2 F, 12 NF
*Breed*	Akita, Border Collie, Cairn Terrier, CKCS (3), Cocker Spaniel, Corgi, Dachshund, Doberman, English Bull Terrier, Golden Retriever, Irish Setter, Labrador (10), Lhasa Apso, Miniature Schnauzer, Mixed Breed (5), Pug (3), Samoyed, Schipperke, Yorkshire Terrier (2)
*Body weight PRE (kg)*	30.6 (5.4 to 77.0)
*Body weight POST (kg)*	23.2 (4.4 to 51.4)
*Body fat mass PRE (g)*	11400 (1600 to 37700); 45% (30 to 55)
*Body fat mass POST (g)*	6300 (700 to 15600); 28% (11 to 45)
*Lean tissue mass PRE (g)*	13800 (3600 to 36600); 52% (43 to 68)
*Lean tissue mass POST (g)*	13300 (3300 to 33400); 68% (53 to 86)
*Duration (days)*	250 (91 to 674)
*Rate of weight loss*^*1*^*(%/week)*	0.8 (0.3 to 1.4)
*Body weight change*^*2*^*(%)*	−28.3 (−16.0 to −40.1)
*Change in fat mass*^*2*^*(%)*	−53 (−78 to −18)
*Change in lean mass*^*2*^*(%)*	−7 (−36 to 10)
*EI during weight loss*^*3*^*(units)*	259 (184 to 310) [62 (44 to 74)]

All data are expressed as median (range). M: male; NM: neutered male; F: female; NF: neutered female; CKCS: cavalier king Charles spaniel. ^1^Rate of weight loss expressed as percentage of starting body weight lost per week. ^2^Refers to the percentage change in starting mass calculated as follows: ([start mass–end mass] ÷ start mass) × 100*%*.^ 3^EI: energy intake expressed as metabolizable energy (in kJ [kcal]) per kg of metabolic body weight (BW^0.75^) per day.

### Changes in clinical biochemistry before and after weight loss

Clinical biochemistry results are shown in Table 3. Urea was greater after weight loss (P = 0.047), whilst albumin (P < 0.001), cholesterol (P = 0.040), globulins (P = 0.012), and triglycerides (P = 0.015) were less after weight loss. However, there was no difference in alanine aminotransferase (P = 0.893), alkaline phosphatase (P = 0.065), and creatinine (P = 0.064) and glucose (P = 0.210).

**Table 3:** Clinical biochemistry results before and after weight loss

***Serum biochemistry***	**Before weight loss**	**After weight loss**	**Reference range**	**P value**
*Alanine aminotransferase (IU/L)*	44 (19–303), 3, 0	52 (18–238), 3, 0	7-100	0.893
*Alkaline phosphatase (IU/L)*	73 (32–383), 7, 0	56 (26–400), 3, 0	0-150	0.065
*Albumin (g/L)*	31 (27–36), 1, 0	29 (26–34), 0, 0	23-35	<0.001
*Cholesterol (mmol/L)*	5.6 (2.5-9.1), 6, 1	5.0 (1.9-7.7), 4, 2	3.5-7.0	0.040
*Creatinine (μmol/L)*	84 (37–123), 1, 0	80 (30–122), 1, 0	20-110	0.033
*Globulins (g/L)*	31 (21–48), 1, 0	27 (22–41), 0, 0	22-40	0.012
*Glucose (mmol/L)*	5.3 (3.5-8.7), 8, 0	5.2 (3.0-7.4), 6, 0	3.5-5.5	0.210
*Urea (mmol/L)*	4.6 (1.6-8.0), 2, 3	5.6 (3.5-8.9), 3, 0	3.5-7.0	0.047
*Triglycerides (mmol/L)*	1.2 (0.6-5.3), 8, 0	0.9 (0.6-2.1), 3, 0	0.6-1.5	0.015

Data are expressed as median (range), number above reference range, and number below reference range. P values quoted for Wilcoxon signed ranks test.

### Changes in plasma nutrient concentrations with weight loss

Plasma nutrient concentrations are shown in Table 4. For the 26 dogs with initial and post sample analysis of choline, betaine, and amino acids, the median time between the two samples (weight loss program duration) was 242 days, with a range of 91–674 days. Plasma amino acid concentrations had wide ranges and no systematic change was noted across all nutrients (i.e. some increased while other decreased with weight loss). Additionally, median concentrations of the amino acid were not outside the reference range of the laboratory (determined by two standard deviations from their median score in 131 healthy animals), with the exception of aspartic acid and glutamic acid, which were more than the reference range before and after weight loss.

**Table 4:** Median (and range) of selected nutrients in dogs pre and post weight loss

***Nutrient***	Mean ± SEM*	**Before weight loss**	**After weight loss**	**P value**
*Choline (umol/L)*	-	68.8 (34.1-287.8)	57.5 (22.9-129.9)	0.046
*Betaine (umol/L)*	-	94.8 (54.2-251.6)	83.0 (44.1-153.4)	0.79
*Urinary selenium (umol/L)*	-	1886 (51–7317)	2633 (430–4634)	0.31
*Urinary Selenium:creatinine*	-	0.10 (0.03-0.20)	0.14 (0.07-0.24)	0.006
*Taurine (nmol/ml)*	77 ± 2.1	99.7 (63.5-232.1)	109.9 (71.0-345.6)	0.19
*Aspartic Acid (nmol/ml)*	7 ± 0.2	14.0 (0.9-27.7)	12.5 (3.6-90.6)	0.73
*Threonine (nmol/ml)*	178 ± 5.0	211.4 (89.6-359.2)	184.5 (85.5-324.6)	0.02
*Serine (nmol/ml)*	107 ± 2.6	110.0 (72.8-168.1)	111.6 (71.0-151.7)	0.64
*Asparagine (nmol/ml)*	40 ± 1.1	37.3 (0.0-63.9)	39.0 (17.6-111.9)	0.68
*Glutamic Acid (nmol/ml)*	23 ± 1.2	154.2 (75.9-303.5)	117.4 (25.2-219.8)	0.07
*Glutimine (nmol/ml)*	-	485.4 (43.7-855.2)	452.3 (170.8-650.7)	0.27
*Glycine (nmol/ml)*	268 ± 8.4	188.6 (101.3-304.9)	204.7 (125.6-290.7)	0.045
*Alanine (nmol/ml)*	388 ± 9.6	373.3 (144.2-783.3)	384.8 (225.8-743.2)	0.99
*Citrulline (nmol/ml)*	41 ± 1.9	47.2 (0.0-159.3)	40.6 (0.0-99.9)	0.08
*Amino-Butyric Acid (nmol/ml)*	-	21.9 (0.0-64.6)	21.7 (5.0-53.3)	0.53
*Valine (nmol/ml)*	157 ± 4.1	186.0 (130.2-275.3)	188.2 (122.9-261.8)	0.52
*Cystathione (nmol/ml)*	-	3.2 (0.0-11.6)	3.8 (1.1-11.0)	0.64
*Isoleucine (nmol/ml)*	51 ± 1.3	67.0 (40.1-96.0)	65.3 (38.0-111.4)	0.23
*Leucine (nmol/ml)*	120 ± 3.2	122.6 (22.6-207.1)	124.8 (62.4-210.3)	0.62
*Tyrosine (nmol/ml)*	39 ± 1.1	38.9 (4.0-71.7)	38.7 (10.6-58.0)	0.49
*Phenylalanine (nmol/ml)*	45 ± 0.9	59.0 (5.6-91.6)	54.2 (20.9-89.6)	0.29
*Ornithine (nmol/ml)*	35 ± 1.5	15.3 (1.8-34.4)	14.5 (1.2-26.5)	0.22
*Lysine (nmol/ml)*	132 ± 5.0	117.7 (11.4-415.8)	93.4 (7.7-251.3)	0.14
*1-Methyl-Histidine (nmol/ml)*	-	6.2 (0.0-27.0)	6.7 (0.0-40.6)	0.82
*Histidine (nmol/ml)*	71 ± 1.6	57.8 (3.5-95.4)	59.0 (10.4-90.4)	0.45
*Tryptophan (nmol/ml)*	60 ±1.7	62.2 (28.7-115.3)	53.2 (19.9-100.0)	0.06
*3-Methyl-Histidine (nmol/ml)*	-	10.5 (4.0-15.9)	9.4 (4.8-19.5)	0.40
*Carnosine (nmol/ml)*	-	29.7 (12.3-49.5)	24.2 (11.5-51.6)	0.09
*Arginine (nmol/ml)*	102 ± 2.6	79.5 (25.8-265.8)	80.9 (15.6-130.0)	0.11
*Hydroxyproline (nmol/ml)*	67 ± 4.1	22.5 (1.1-52.1)	22.0 (0.0-103.7)	0.24
*Proline (nmol/ml)*	246 ± 8.2	140.0 (80.4-234.0)	123.6 (85.3-327.6)	0.79

* References ranges taken from Delaney et al., 2003 [21].

Plasma concentrations of choline (*P* = 0.046) and threonine (*P* = 0.02) both decreased with weight loss. In contrast, plasma glycine concentration (*P* = 0.045) and urinary selenium:creatinine ratio (*P* = 0.006) both increased. There were no significant changes for any of the other plasma nutrient concentrations. Further, none of changes observed were associated with alterations in clinical biochemistry parameters (Kendall’s tau −0.15 to 0.28, P > 0.1 for all), and there was no association between changes in choline and betaine concentrations before and after weight loss (Kendall’s tau −0.18, P = 0.203).

### Associations between weight loss outcomes and changes in plasma nutrient concentration

Simple and multiple linear regression analyses were used to determine factors associated with the significant changes in nutrients identified. For changes in plasma choline concentration, simple linear regression identified possible associations (at *P* < 0.2) for breed group, starting body fat percentage, starting lean tissue mass, and rate of weight loss (Table 5). However, two models, with equally good fit were identified (Table 6). For model 1, starting lean tissue mass (i.e. greater starting lean mass, greater decrease in plasma choline concentration; *r* = −0.47, *r*^*2*^ = 0.22, *P* = 0.02) and duration of weight loss (i.e. longer duration of weight loss, greater decrease in plasma choline concentration; *r* = 0.35, *r*^*2*^ = 0.12, *P* = 0.08) were retained in the model. For model, 2 starting lean tissue mass was again included (i.e. greater starting lean mass, greater decrease in plasma choline concentration; *r* = −0.45, *r*^*2*^ = 0.20, *P* = 0.02), but instead with rate of weight loss (i.e. slower rate of weight loss, greater decrease in plasma choline concentration; *r* = 0.35, *r*^*2*^ = 0.12, *P* = 0.08; Table 3). This was because there was a strong negative association between duration of weight loss and rate of weight loss (Kendall’s tau −0.67, *P* < 0.001).

**Table 5:** Simple linear regression to determine factors associated with changes in percentage plasma choline concentration

**Simple regression**	**Regression coefficient**	**r**	**r**^**2**^	***P***
Age [months]	0.2	0.26	0.07	0.204
Sex	10.8	0.18	0.03	0.364
Neuter status	14.5	0.13	0.02	0.518
Breed group^*^	−23.8	−0.40	0.16	0.043
Starting weight [kg]	−1.0	−0.57	0.32	0.002
Body fat percentage (pre) [%]	−2.3	−0.48	0.23	0.014
Lean tissue mass (pre) [kg]	−1.9	−0.54	0.29	0.004
Duration of weight loss [days]	−0.09	−0.46	0.21	0.018
Rate of weight loss [%SBW/week]	42.4	0.48	0.23	0.013
Percentage weight loss [%]	0.1	0.03	0.00	0.873
Energy intake during weight loss	−0.6	−0.14	0.02	0.493
Change in fat tissue mass [%]	−0.5	−0.19	0.04	0.345
Change in lean tissue mass [%]	−0.02	0.00	0.00	0.979

^*^ Breed based upon a dummy variable where dogs of retriever breeds were assigned a value of 1.

**Table 6:** Multiple linear regression to determine factors associated with changes in percentage plasma choline concentration

**Model 1**	**Regression coefficient**	**r**	**r**^**2**^	***P***
Final model		0.62	0.33	0.004
Lean tissue mass (pre) [kg]	−1.5	−0.47	0.22	0.019
Duration	−0.06	−0.35	0.12	0.082
**Model 2**	**Regression coefficient**	**r**	**r**^**2**^	**P**
Final model		0.62	0.33	0.004
Lean tissue mass (pre) [kg]	−1.5	−0.45	0.20	0.025
Rate of weight loss	28.4	0.35	0.12	0.082

For plasma glycine concentration, simple linear regression identified an association with breed group (i.e. greater gain in glycine concentration in non-retriever breeds; *r* = −0.40, *r*^*2*^ = 0.16, *P* = 0.045) only. However, none of the factors tested were found to be significantly associated with plasma threonine concentration, or urinary selenium:creatinine ratio (*P* > 0.1 for all, full data not shown).

## Discussion

In the current study, changes in plasma or urinary nutrients have been assessed before and after a controlled weight loss plan. Changes in plasma and urinary status nutrient status are the result of a combination of factors including the requirement of the individual dog for that nutrient, the level of inclusion in the diet, and daily food consumption. If a diet is used to induce safe weight loss in obese dogs, nutrients are included at increased amounts relative to the energy content of the diet, to minimise the risk of a deficiency occurring when energy intake is restricted [34]. Therefore, the extent to which the process of formulation is successful is reflected in changes in nutrient status, or lack thereof. A widespread decline in nutrient status was not seen although plasma or urinary concentrations of certain nutrients, most notably choline, did decline significantly. This suggests that choline requirements might increase during caloric restriction, especially in larger dogs (i.e. with a greater lean body mass) and those dogs requiring a longer duration weight loss program. Other changes included a decrease in plasma threonine concentration, and increases in plasma glycine concentration and urinary selenium:creatinine ratio. Therefore, although the results should be considered in light of the limitations of the study, the status of certain nutrients should be carefully reviewed in dogs during caloric restriction. All dogs in the current study were given the same purpose-formulated weight loss diet and, as a result, the findings cannot necessarily be generalized to other diets used for weight management. Therefore, additional studies are now warranted, assessing a wider range of diets fed at different levels of caloric energy intake. Further, the risks of deficiency are likely to be greater if a diet formulated for maintenance were used instead of a purpose-formulated weight loss diet, where the nutrient content is supplemented relative to energy content.

The dogs remained healthy during their period of weight loss, and did not demonstrate any signs of deficiency. A minority (<5%) of plasma biochemical parameters were outside the reference ranges both before and after weight loss. This is consistent with what would be expected by chance since reference intervals are typically established to include ~95% of the normal canine population [35]. Further, although some biochemical parameters changed during weight loss (either increasing or decreasing), the majority of results remained within the reference interval. Indeed, these findings are the same as those that have been reported previously [25,33,36]. This would suggest that such changes are not of clinical significance. Furthermore, the changes in these biochemical parameters are unlikely to be associated with the observed changes in nutrient status because there was no significant association between them.

With the exception of threonine and glycine, there were no significant changes in any plasma concentrations of essential and non-essential amino acids in this study. Since each amino acid has a unique role in the body, and particular pathways for synthesis and degradation, there may be various reasons for this. Firstly, the diet used for weight loss was supplemented in protein relative to its energy content and, thus, able to meet minimum requirements even during a period of caloric restriction. Further, although the plasma concentration of threonine decreased during weight loss, concentrations for all dogs remained within the laboratory’s reference range, no signs of deficiency were seen in any dog, and the changes did not correlate with lean tissue loss during weight loss. This suggests that a clinical deficiency was unlikely. In contrast, plasma glycine concentration increased with weight loss although, once again, all concentrations remained within the laboratory’s reference range.

Previous studies have indicated that methionine is susceptible to oxidation during storage [30], whilst cysteine is unstable in storage if samples are not deproteinised by treating with sulphosalicylic acid immediately after sampling [31]. Given that the storage conditions of the current study were not optimized for either amino acid, we were concerned that results would not be reliable and, therefore, chose not to analyse them. This is a potential limitation, not least in light of a previous *in silico* study suggesting that both might be at risk of deficiency during caloric restriction [13]. As a result, a further study should be considered to assess these nutrients in dogs undergoing caloric restriction, using samples that have been appropriately prepared and analysed. Unexpectedly, median concentrations of glutamic acid and aspartic acid were greater than the laboratory range in many dogs, and the reasons for this are again not known. An issue with sample storage or subsequent assay is possible, and further assessment should now be considered.

Selenium is a mineral involved in antioxidant pathways and thyroid function, and is important for immune system function [26]. Selenium status is tightly regulated in dogs, and many different methods of assessment have been suggested, including measurement of plasma selenium, blood selenium, selenoproteins, erythrocyte glutathione peroxidase, urinary selenium and liver selenium [14,36]. The lack of a single ‘gold-standard’ assay method highlights the difficulty in accurate assessment of selenium status in dogs. In the current study, increased urinary selenium excretion was noted after the period of dietary restriction, which is notable in light of previous work demonstrating that urinary selenium excretion does not vary greatly with different diets [36]. The reasons for this finding are not known. First, urine selenium:creatinine ratio was used as a proxy measure, for 24-hour urinary excretion, and this ratio has been validated against 24 h selenium excretion in humans [37]. Whilst this measure has not been validated in dogs, urinary ratios based upon creatinine are widely accepted for measurement of other substances such as protein [38] and cortisol [39]. Second, only 20 pairs of urine samples were tested, and these might not have been representative. Against this, however, was the fact that there were no differences in either baseline parameters or weight loss outcomes in the dogs where urinary selenium was measured, compared with those not tested. Third, it is possible that, despite caloric restriction, selenium intake on the weight loss diet was greater than on the baseline diets. Unfortunately, owners of the study dogs had not maintained accurate records of food intake prior to weight loss, making it difficult to determine if this was, indeed, the case. Given it was not possible to assess selenium status with additional methods, such as erythrocyte glutathione peroxidase or measurement of selenoproteins [14], these results should be interpreted cautiously and need confirmation in future studies.

Whatever the reason, these findings refute concerns highlighted in a recent *in silico* study that suggested selenium intake might be deficient when fed using the same weight loss diet at the predicted levels of caloric restriction [13]. This might be due to the fact that the calculations in the previous study were based upon dietary requirements recommended by the NRC [26], but the actual selenium requirements of adult dogs are not known, partly due to the difficulty in accurate assessment of selenium status in animals. Further, it is possible that requirements decrease during a period of underfeeding, as would be encountered on a weight loss program. Additional studies that can better assess selenium status in relation to healthy dogs are now warranted, for example by measuring blood and liver selenium concentrations in dogs undergoing different degrees of caloric restriction.

Choline is a vitamin-like substance that has multiple roles in the body, including neurotransmission, hepatic lipid transport and storage, coagulation, and as a methyl donor as a part of phosphatidylcholine [26]. Amongst five diets analysed in a previous *in silico* study [13], choline was the second most likely nutrient to be at risk of deficiency during caloric restriction. All dogs in the current study received a diet containing of 860 mg choline per 1000 kcal metabolisable energy and, fed at this concentration, intake might be less than NRC recommended allowance. For instance, the daily choline intake of an obese dog with a target weight of 23.2 kg, receiving a daily energy intake of 62 kcal/kg^0.75^ (the median energy intake during weight loss for the current study), would be 563 mg, which is 95% of the recommended daily choline allowance (592.0 mg/day) [26]. The NRC recommended allowances (RA) are not minimum requirements (MR), or ‘the minimal concentration of a maximally bioavailable nutrient that will support a defined physiological state’ [26], and intake less than RA will not necessarily lead to a deficiency state. During the NRC review, when the MR of a particular nutrient was known, the NRC RA was estimated, for instance being based upon adequate intake (AI) or ‘the concentration of nutrient demonstrated to support a defined physiological state, when no MR has been demonstrated’ [26]. Not surprisingly given the way it is determined, AI is usually greater than MR. In the case of choline, the current RA was based on AI data from studies conducted over 50 years ago [26]. In light of this, the significance of the decrease in plasma choline concentration seen in the current study is not known.

For the changes in choline status to be of significance, clinical signs of choline deficiency would be expected. In dogs, choline deficiency has been associated with hypocholesterolaemia, vomiting, fatty liver disease, and death [14]. However, all dogs remained clinically well throughout the study and did not demonstrate any of these signs, or any signs that might be associated with liver disease (e.g. icterus). Further, there were no changes in liver enzyme activity and, although, serum cholesterol decreased, none of the dogs had concentrations less than the reference range after weight loss. Thus, it is unlikely that the changes in choline status were clinically relevant. One additional factor we considered as a cause of the observed changes in choline was plasma betaine status. Choline is metabolised to betaine, by the sequential action of choline dehydrogenase and betaine aldehyde dehydrogenase [40]. However, the fact that there were no changes in plasma betaine with weight loss, and no association with changes in plasma choline concentrations, suggests that this was not the reasons for the differences observed in the current study.

As with selenium, there is a lack of published information on choline status in dogs and, in particular, methods of determining deficiency. In a previous study [41] total choline and phospholipid-containing choline concentrations were measured using the protozoa *Torulopsis pintolopessi*, a method was previously described in rats and man [42]. Their results were reported in μl/mL and, when converted to μmol/L, the median choline concentration was 4128 μmol/L (range: 2255–7678) in normal dogs. These results are different from those of the current study both before (median 68.8 μmol/L; range: 34.1-287.8) and after (57.5 μmol/L: range: 22.9-129.9) weight loss. However, more technologically advanced methods were used in the current study, including mass spectrometry, which is the gold standard method in human medicine [27]. This difference in methodology, and the previous study including not only choline, but also all phospholipid-containing choline, could explain the difference in results. Given this difference, further studies using similar methodology are warranted to determine choline status in healthy dogs. Signs of choline deficiency in dogs, cats and humans can include weight loss, hepatic lipidosis, anorexia, vomiting, neurological signs, disorders, and coagulopathies [26,43]. However, even though plasma choline concentration decreased during weight loss in the current study, the dogs were regularly re-evaluated throughout their period of weight loss, did not display any clinical signs consistent with choline deficiency. That said, the possibility of a subclinical choline deficiency cannot be excluded. However, it is also possible that either endogenous choline production or dietary intake of other nutrients might alter or lessen the dietary requirements or that choline requirements decrease during periods of energy restriction. In diet formulation, choline is relatively easy nutrient to supplement, and there are minimal toxicity concerns. For this reason, further studies assessing benefit of supplementation of choline during weight loss programs are warranted.

A number of limitations should be considered when interpreting the results of the current study. First, we chose to study a group of client-owned dogs with naturally-occurring obesity, and this group was diverse in terms of the diet fed before weight loss, environment and housing, and signalment. The degree of excess adiposity also varied, as did the caloric restriction required to achieve weight loss, and the rate of weight loss achieved. Further, as mentioned above, due to ethical limitations we were not able to include a control group, for instance, a population of obese dogs undergoing weight loss using a standard maintenance diet. Ethical limitations were also the reason why plasma and urine nutrient measurement was chosen, instead of tissue biopsy for instance, as the principle means of assessing nutrient status. Whilst such a group would have undoubtedly strengthened our findings, use of such a group would not be allowed in a clinical study involving pet dogs and, instead, laboratory dogs would have had to be used. However, the advantage of using client-owned animals, with naturally occurring obesity, was the fact that the findings are representative of the true clinical picture. Furthermore, measuring at two time points, enabled dogs to act as their own controls, effectively controlling for many individual differences, namely baseline parameters and environment. A second limitation was the fact that dogs were on a variety of diets prior to starting the weight loss program, and exact details (i.e., products used and amounts fed) were lacking. Thus, the differences identified before and after weight loss might instead reflect differences in dietary composition rather than being the result of caloric restriction, *per se*.

A third limitation was the fact that it was not possible to conduct all nutrient analyses for all dogs. In this respect, selenium analyses were only conducted on urine samples from a subset of the study dogs, whilst five plasma samples were lost when their tubes were damaged in transit. Although this might have influenced the results obtained, these subsets were broadly similar to the main study population. Given that fewer sample pairs were tested than was planned, study power is likely to be less than anticipated for these measurements. However, the fact that differences in both plasma choline and urinary selenium were identified, suggests that the study findings were not materially affected. Also, on the issue of study power, a fourth limitation was the fact that, since a study of this nature had not been performed previously, it was not possible to perform a meaningful *a priori* power calculation. Given that differences before and after weight loss were not identified for many nutrients, it is possible that some statistical comparisons were underpowered. Nonetheless, if necessary, the results of the current study can now be used to determine study numbers required for a future study.

A final study limitation was the fact that, whilst owners were instructed to feed the weight loss diet exclusively, some owners still gave their dogs additional food, in the form of treats and table scraps. Although these extra foodstuffs make a minor contribution to overall intake, on average 1% of MER [11], these may nonetheless, have had an impact on results. Unfortunately, such foods were not recorded accurately in the current study.

## Conclusions

The current study has suggested that plasma or urinary concentrations for many nutrients do not change appreciably when obese dogs undergo a weight loss program. The main exceptions were decreases in plasma choline and threonine concentrations, and increases in plasma glycine concentration and urinary selenium:creatinine ratio. The reason for these changes are not fully clear and results should be considered in light of the limitations of the study. Nonetheless, the significant decrease in plasma choline concentration during weight loss might suggest increased choline requirements during weight loss. Despite this decrease, no signs of choline deficiency were noted during the weight loss program, suggesting that clinical consequences did not occur, although subclinical nutrient deficiency cannot be excluded entirely. Further studies are now warranted to assess a wider range of diets, fed at different levels of caloric restriction.

## Abbreviations

DEXA: Dual-energy x-ray absorptiometry; MER: Maintenance energy requirement; NRC: National research council; RER: Resting energy requirement; SBW: Starting body weight.

## Competing interests

The following conflicts of interest apply: AJG’s Senior Lectureship is funded by Royal Canin; the diet used in this study is manufactured by Royal Canin; VB is employed by Royal Canin.

## Authors’ contributions

DL – designed the study, liaised with laboratories and oversaw sample handling, drafted the manuscript, and reviewed the manuscript; LF - designed the study, reviewed the results, and reviewed the manuscript; SLH – collected clinical data, and reviewed manuscript; VB – designed the study, reviewed the results, and reviewed the manuscript; AJG – designed the study, collected the clinical data, analysed the results, and drafted the manuscript. All authors have approved the final article.
